# Photo-Protective Mechanisms and the Role of Poly (ADP-Ribose) Polymerase Activity in a Facultative CAM Plant Exposed to Long-Term Water Deprivation

**DOI:** 10.3390/plants9091192

**Published:** 2020-09-12

**Authors:** Luca Vitale, Ermenegilda Vitale, Giulia Costanzo, Anna De Maio, Carmen Arena

**Affiliations:** 1 National Research Council (CNR), Department of Biology, Agriculture and Food Science (DiSBA), Institute for Agricultural and Forestry Systems in the Mediterranean (ISAFoM), P.le E. Fermi 1-Loc. Porto del Granatello, 80055 Portici (Na), Italy; 2 Department of Biology, University of Naples Federico II, Via Cinthia, 80126 Naples, Italy; ermenegilda.vitale@unina.it (E.V.); giul.costanzo@studenti.unina.it (G.C.); andemaio@unina.it (A.D.M.)

**Keywords:** CAM, CAM-idling, oxidative stress, enzymatic antioxidants, PARP, photochemistry, photorespiration, *P. afra*

## Abstract

The Crassulacean acid metabolism (CAM) pathway helps plants to alleviate the oxidative stress under drought, but the shift to CAM-idling may expose plants to the overproduction of reactive oxygen species causing cell damages. The facultative CAM species *Portulacaria afra* L., was subjected to long-term water deprivation to assess the photo-protective strategies and the poly (ADP-ribose) polymerase (PARP) activity during water stress and plant capability to recover from the stress. Measurements of titratable acidity, chlorophyll fluorescence emission, and antioxidant activity were performed during the stress and rewatering. Under water deprivation, plants shifted from C3 to CAM metabolism, reaching the CAM-idling status at the end of the stress period. The daily variation of the titratable acidity and PARP activity increased at the beginning of stress and declined with stress progression, reaching the lowest value at the end of stress treatment. H_2_O_2_ content, superoxide dismutase (SOD), peroxidase (POD), and catalase (CAT) activities increased with the severity of water stress. The photochemical processes remained high during the entire stress period indicating the presence of alternative sinks to CO_2_ fixation. The elevated activity of catalase under severe water stress suggests the occurrence of photorespiration in sustaining the photosynthetic electron transport under CAM-idling condition. The overall data indicate that scavenger enzymes, photorespiration and PARP activity modulation contribute to the strong resistance of *P. afra* to severe water stress, preserving the functioning of photosynthetic apparatus and ensuring plant recovery with rewatering.

## 1. Introduction

Crassulacean acid metabolism (CAM) represents a carbon fixation pathway typical of plants adapted to arid environments. In CAM plants the stomata are open during night and the CO_2_ uptake occurs with organic acids synthesis (viz. malate). The CO_2_ fixation is mediated by phosphoenolpyruvate carboxylase, PEPC (phase I). In the morning, during the early light period the simultaneous CO_2_ fixation by PEPC and Rubisco occurs while stomata start to close (phase II). During the day, stomata are closed, and organic acids stored during night are decarboxylated. The released CO_2_ is re-fixed by Rubisco and assimilated in C3 pathway (phase III). In the late light period, stomata reopen and the CO_2_ uptake as well as the carbon fixation by Rubisco starts again (phase IV). In the last phase, a photorespiratory carbon flux occurs.

CAM may operate in different ways. In the obligate CAM plants, a high nocturnal acid accumulation and diurnal CO_2_ re-fixation occur. In the facultative or inducible CAM plants, the photosynthetic pathway shifts from C3 or C4 to CAM in response to photoperiod length or drought. In severe water-stress conditions, stomata may remain closed during the entire day and a small nocturnal acid accumulation occurs due to the recycling of the CO_2_ produced by respiration. This condition is known as CAM-idling.

It has been demonstrated that CAM may help to alleviate the oxidative stress in response to environmental constraints by consuming the reductive power of the photosynthetic electron transport chain in CO_2_ assimilation during phase III [1]. During this phase, CAM plants are exposed to oxidative stress [2], due to the formation of reactive oxygen species (ROS) as a consequence of the high O_2_ production in the light [3,4]. The excess of ROS may be dangerous for cells, causing DNA damages and, in extremis, cell death (i.e., necrosis or apoptosis). Both facultative and constitutive CAM plants can limit the oxidative stress promoting the activity of the xanthophyll cycle [5,6] and antioxidant enzymes (i.e., ascorbate peroxidase, superoxide dismutase, glutathione reductase, and monodehydroascorbate reductase) [7,8,9], as well as the accumulation of γ-tocopherol [10]. During phase IV, a photoprotective role of photorespiration has also been proposed for CAM plants, even if it would be less important compared to the CO_2_-concentrating mechanism operating in the phase III [11]. Nevertheless, under prolonged water stress the photorespiration pathway might become operational in the CAM-idling status due to low CO_2_ levels during the day as consequence of the reduced nocturnal acid accumulation. Previous studies showed that the activity of photorespiratory enzymes remained high in obligate and facultative CAM species [7,12].

An unexplored hypothesis is represented by the possibility that under severe water stress, CAM as C3 plants may counteract the elevated levels of ROS production by the overactivation of poly (ADP-ribose) polymerase enzymes (PARPs). PARPs play an important role in various cellular processes, including modulation of chromatin structure, transcription, replication, recombination, and DNA repair. Although PARP enzymes have been found to operate in C3 plants under different stress conditions [13,14,15,16], to date no study has been carried out on the relationship between CAM plants and PARP activity under water stress. The shift of CAM to CAM-idling, in response to intense water stress, may trigger the ROS overproduction in plants [6,17], exposing cells to elevated risks of oxidative stress and DNA damages. These circumstances could activate a prompt response by PARP enzymes, engaged in the repair of possible DNA damages.

In the past, studies on obligate or facultative CAM plants have been mainly focused on the modulation of photosynthetic metabolism and photo-protective mechanisms in response to short-term water deprivation, while no information is available under long-term water deprivation, a stress condition that should induce a CAM-idling status.

*Portulacaria afra* L. Jacq. is a native South Africa perennial succulent shrub showing a seasonal shift from C3 to CAM metabolism [18] in response to long days [19] or water stress [20]. This species represents a suitable study model because it utilizes the transition from CAM to CAM-idling to endure long drought periods, and it is capable of quick recovery following rewatering. Previous studies demonstrated that the ability of *P. afra* to restore from the stress is due to the maintaining of activity of Rubisco carboxylase, PEP carboxylase, and PEP carboxykinase enzymes [20]; however, further physiological reasons could be responsible for the elevated resistance of *P. afra* to drought. In this study, we evaluated the occurrence and extent of photo-protective strategies in *P. afra* plants during the transition from C3 to CAM and from CAM to CAM-idling induced by the severe and prolonged water stress conditions. We also analyzed, for the first time, the modulation of the PARP activity and the putative role of photorespiration in limiting the occurrence of oxidative damages in phase III under CAM-idling status.

## 2. Results

### 2.1. Leaf Water Status and Photosynthetic Pigment Content

The relative water content, RWC, decreased with the increase of the water stress (Table 1). The lowest value (47%) (*p* < 0.01) was reached at 79 Days Without Water (DWW) and remained constant (47%) up to 94 DWW. With plant rewatering, RWC full recovered, returning to pre-stress values (89%). Photosynthetic pigment content (total chlorophyll and carotenoids), was not affected by water stress and remained almost constant during the whole stress period and plant rewatering, ranging from 30.1 ± 1.1 and 38.7 ± 2.0 μg cm^−2^ for total chlorophylls, and 7.5 ± 0.3 and 9.1 ± 0.4 μg cm^−2^ for total carotenoids.

### 2.2. Titratable Acidity Fluctuation

**Figure 1:**
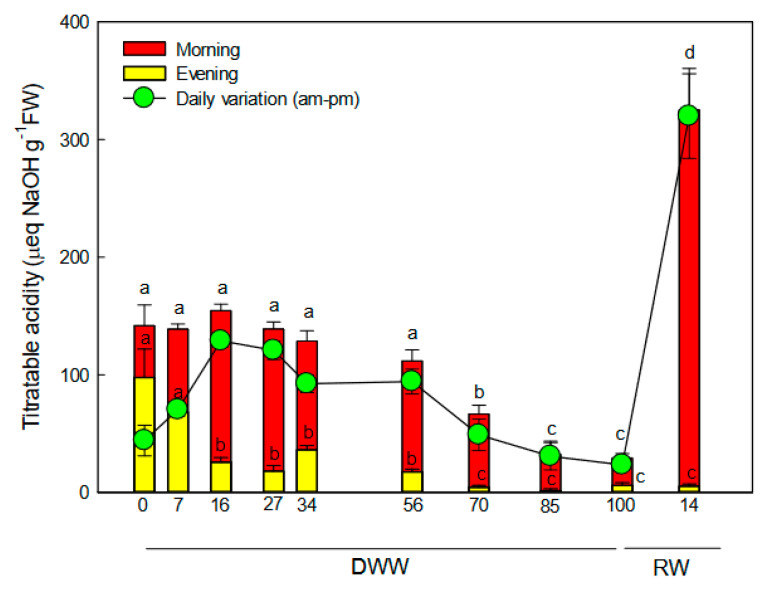
Trend of daily acid fluctuation during days without water (DWW) and rewatering (RW). Different letters indicate statistically significant differences in morning or evening acid content (*p* ≤ 0.05). Data were analyzed by one-way ANOVA repeated measurements followed by the Tukey’s test. Data are means (*n* = 10) ± SE.

Before the water stress, plants showed a small fluctuation in titratable acidity from morning to night, suggesting that the C3 cycle was the main functioning photosynthetic pathway in *P. afra* and that limited CAM activity (likely CAM-cycling) occurred too (Figure 1). Under stress, *P. afra* plants showed a large fluctuation of titratable acidity from morning to night (*p* < 0.01). The titratable acidity variation peaked (120 μeq NaOH g FW^−1^) at 15 DWW, as the consequence of the increased daytime organic acid degradation produced and accumulated during the night (Figure 1). Our data suggest an active CAM pathway in this circumstance. With the progression of water stress, the fluctuation of titratable acidity decreased (*p* < 0.01) for the reduction of the nocturnal acid accumulation, reaching the lowest value (20–30 μeq NaOH g^−1^ FW) at 100 DWW. At this time *P. afra* plants seem to reach the CAM-idling status. With rewatering, the acid accumulation during the night significantly raised, determining a marked increase of daily variation of titratable acidity, which suggested that the CAM pathway was fully active.

### 2.3. Fluorescence Measurements

**Figure 2:**
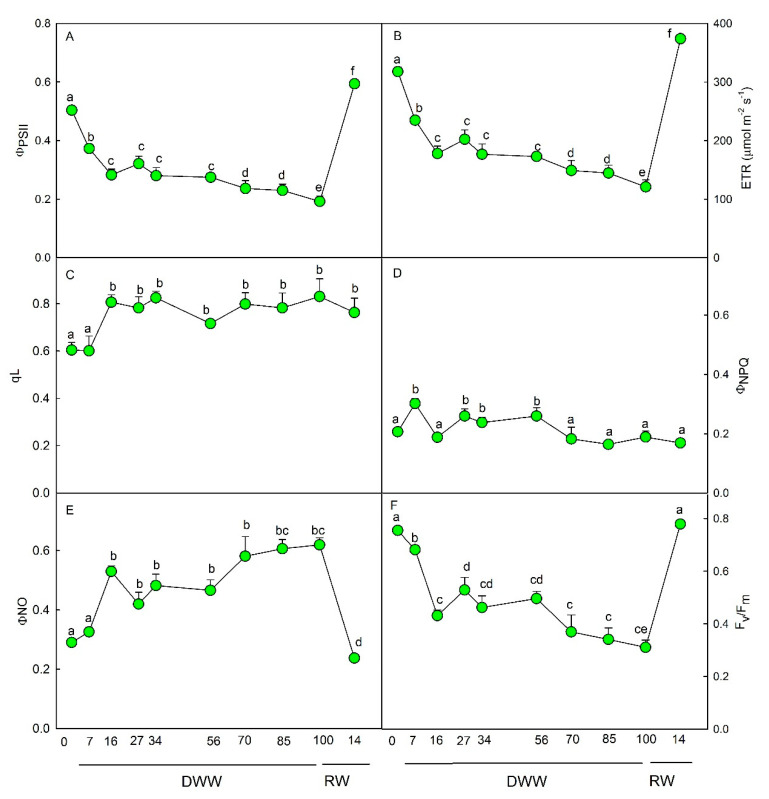
Trends of (**A**) quantum yield of photosystem II electron transport, ΦPSII; (**B**) electron transport rate, ETR; (**C**) photochemical quenching, qL; (**D**) yield of regulated energy dissipation, ΦNPQ; (**E**) yield of non-regulated energy dissipation, ΦNO; (**F**) maximal PSII photochemical efficiency, F_v_/F_m_, during days without water (DWW), and rewatering (RW). Different letters indicate statistically significant differences (*p* ≤ 0.05) among different days. Data were analyzed by one-way ANOVA repeated measurements followed by the Tukey’s test. Data are means (*n* = 10) ± SE. Photochemical indexes have been extracted at 1500 Photosynthetic Photon Flux Densities (PPFD) by rapid light curve.

The quantum yield of photosystem II electron transport (Φ_PSII_) (Figure 2A) and the electron transport rate (ETR) (Figure 2B) progressively decreased from 0 to 16 DWW (*p* < 0.01). With the progression of water stress, no further significant decline of Φ_PSII_ and ETR was observed compared to the value reached at 16 DWW.

The photochemical quenching, qL, raised at 16 DWW (*p* < 0.01) and remained almost constant for the remaining DWW (Figure 2C). The quantum yield of regulated energy dissipation (Φ_NPQ_) increased few days after the imposition of water stress up to 60 DWW, then it decreased to pre-stress values (Figure 2D). The quantum yield of non-regulated energy dissipation (Φ_NO_) progressively raised with the severity of water stress, reaching the highest value (*p* < 0.01) at 80 and 100 DWW (Figure 2E). On the contrary, the maximal PSII photochemical efficiency, F_v_/F_m_, gradually declined (*p* < 0.01) as water stress increased. The lowest value of F_v_/F_m_ was observed at 100 DWW (Figure 2F). Measurements carried out 14 days after plant rewatering showed the recovery of all photochemical parameters compared to pre-stress conditions (0 DWW) except for qL, which remained higher and Φ_NO_, which stayed lower compared to 0 DWW (Figure 2).

### 2.4. H_2_O_2_ Content, Non-Enzymatic and Enzymatic Antioxidants

**Figure 3:**
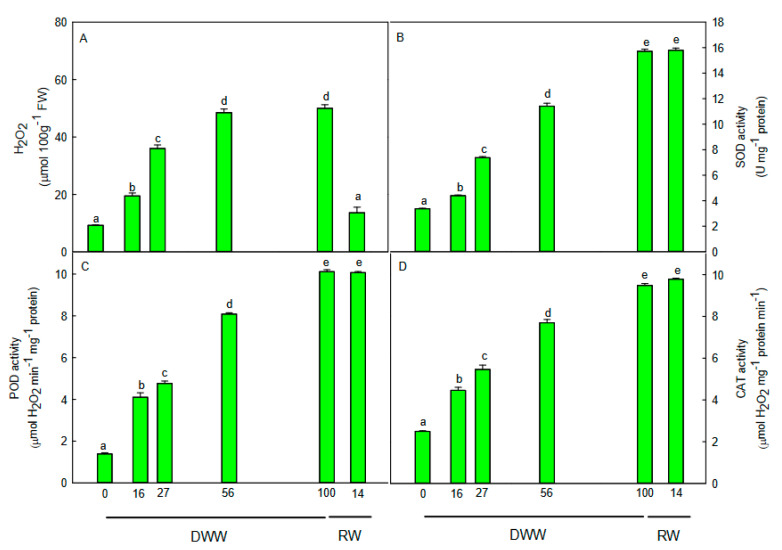
Trends of (**A**) H_2_O_2_ content, (**B**) superoxide dismutase (SOD) activity, (**C**) peroxidase (POD) activity, and (**D**) catalase (CAT) activity during days without water (DWW), and rewatering (RW). Different letters indicate statistically significant differences (*p* ≤ 0.05) among different days. Data were analyzed by one-way ANOVA repeated measurements followed by the Tukey’s test. Data are means (*n* = 10) ± SE.

The progressive water deprivation determined a significant increase (*p* < 0.01) of the leaf hydrogen peroxide (H_2_O_2_) content (Figure 3A). The maximum concentration was found at 56 DWW. At 100 DWW; no further increase of H_2_O_2_ level was measured compared to 56 DWW.

**Figure 4:**
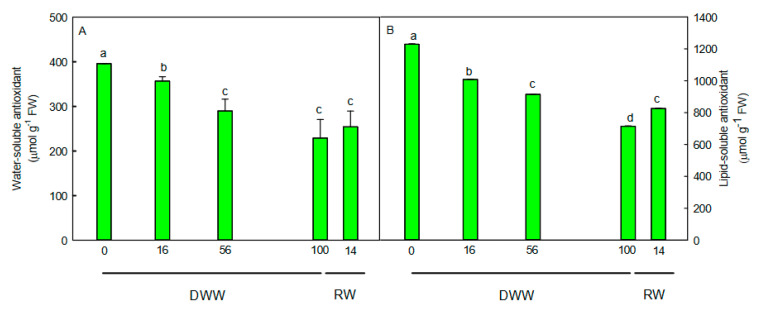
(**A**) water-soluble and (**B**) lipid-soluble antioxidants during days without water (DWW), and rewatering (RW). Different letters indicate statistically significant differences (*p* ≤ 0.05) among different days. Data were analyzed by one-way ANOVA repeated measurements followed by Tukey’s test. Data are means (*n* = 10) ± SE.

The enzymatic (Figure 3B–D) and non-enzymatic (Figure 4) antioxidants exhibited a different trend. The activity of the antioxidant enzymes superoxide dismutase (SOD), peroxidase (POD), and catalase (CAT) significantly increased (*p* < 0.01) just after a few days from water stress imposition compared to pre-stress condition (Figure 3B–D), showing a maximum (*p* < 0.01) of activity at 100 DWW. With the rewatering, the H_2_O_2_ content decreased to pre-stress values, while the antioxidant enzyme activity remained higher and comparable to that measured at 100 DWW (Figure 3).

On the contrary, the water-soluble (Figure 4A) and lipid-soluble (Figure 4B) antioxidant capacity decreased as water stress increased, showing a reduction of 42% (*p* < 0.01) at 100 DWW compared to 0 DWW. With rewatering, the water-soluble and lipid-soluble antioxidant capacity did not recover to pre-stress values but remained lower. In particular, the water-soluble antioxidant capacity did not change, while the lipid-soluble antioxidant capacity weakly increased (*p* < 0.01) compared to values measured at 100 DWW (Figure 4).

### 2.5. PARP Activity

**Figure 5:**
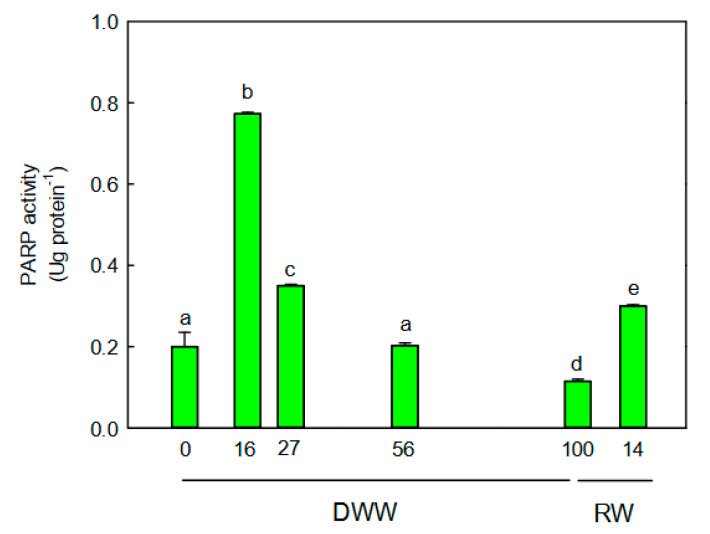
Trends of Poly (ADP-Ribose) Polymerase (PARP) activity during days without water (DWW), and rewatering (RW). Different letters indicate statistically significant differences (*p* ≤ 0.05) among different days. Data were analyzed by one-way ANOVA repeated measurements followed by Tukey’s test. Data are means (*n* = 10) ± SE.

The PARP activity showed a similar trend compared to that observed for the daily fluctuation of titratable acidity (Figure 5). More specifically, the PARP activity peaked at 15 DWW (*p* < 0.01) and progressively declined until 100 DWW, when plants were likely to be found in CAM-idling status. The lowest enzymatic activity was measured at 100 DWW. With rewatering, the PARP activity significantly increased (*p* < 0.001) compared to values measured at 0 and 100 DWW (Figure 5).

### 2.6. Correlations among Investigated Parameters

The correlation among the investigated parameters were reported in Table S1. Φ_PSII_ showed a negative correlation with Φ_NO_ (r = −0.968) and H_2_O_2_ (r = −0.815) and a positive correlation with F_v_/F_m_ (r = 0.974). Φ_NO_ was negatively correlated to ETR (r = −0.968) and F_v_/F_m_ (r = −0.994). SOD was positively correlated to POD (r = 0.980) and CAT (r = 0.987) and negatively correlated to water-soluble (r = −0.988) and lipo-soluble (r = −0.967) antioxidants. POD exhibited a positive correlation with CAT (r = 0.997) and a negative correlation with water-soluble (r = −0.983) and lipo-soluble (r = −0.970) antioxidants. CAT was negatively correlated to water-soluble (r = −0.975) and lipo-soluble (r = −0.968) antioxidants. Water-soluble and lipo-soluble antioxidants showed a positive correlation each other (r = 0.973).

## 3. Discussion

The CO_2_ concentration and the stomatal closure in phase III during the day result in the increase of water use efficiency in CAM plants but also in a potential oxidative stress, due to the sustained photosynthetic electron transport and the rise of internal O_2_ concentration [21]. This condition is particularly dangerous under CAM-idling status, when gas exchanges are suppressed, the accumulation and decarboxylation of organic acids (i.e., malate/citrate) as well as the CO_2_ fixation by Rubisco are reduced. In our experiment, the C3 pathway was the main functioning photosynthetic pathway in unstressed *P. afra* plants, while the reduced daily acid fluctuation is attributable to the CAM-cycling operating with absence of nocturnal CO_2_ uptake [20].

When exposed to water stress, plants likely shifted to CAM right after water stress imposition and to CAM-idling at the end of the stress period (80–100 DWW). This behavior is suggested by the daily fluctuation in titratable acidity, which showed the lowest value (30 μeq NaOH g^−1^ FW) at 80–100 DWW, in concomitance with the lowest leaf RWC. Daily measurement of gas exchanges could have confirmed the evidence of both CAM and CAM-idling status in *P. afra* plants in response to prolonged water stress. However, even if we could not measure the gas exchanges, our hypothesis is supported by the recent work of Guralnick et al. [22], which demonstrated the development of CAM activity through the titratable acidity levels and diurnal acid fluctuations. The daily acid fluctuation measured in the present study is in agreement with values found by Guralnick and Ting [20] in *P. afra* plants exposed to prolonged water stress. These authors report values of daily fluctuation of titratable acidity of 40–50 μeq NaOH g^−1^ FW and no nocturnal gas exchange in stressed plants operating in CAM-idling mode. Based on this evidence, we hypothesized that *P. afra* plants at 100 DWW were in CAM-idling mode, while they moved to full CAM after rewatering under long photoperiod [20], on the basis of the greatest daily acid fluctuation measured after 14 days from water supply.

In our experimental conditions, endogenous H_2_O_2_ content increases as water stress progresses, suggesting the occurrence of oxidative stress, even if the possibility that H_2_O_2_ may act also as a signal involved in C3-CAM transition cannot be excluded [23]. In response to H_2_O_2_ production, the activity of SOD, CAT, and POD scavenger enzymes promptly rose and remained high during the period of water stress and recovery. Our data agree with other studies that found an increase of antioxidant enzyme activity during the C3-CAM transition in response to mild water stress [7,24]. The unexpected result is the high level of CAT activity at 100 DWW when *P. afra* shifted likely to CAM-idling [24]. We suppose that the sustained CAT activity during the exacerbated water stress was effective in removing the H_2_O_2_ also produced in the photorespiration [25]. Generally, catalase in peroxisomes neutralizes H_2_O_2_ into O_2_ and H_2_O [26], and its activity decreases when photorespiration is suppressed [27]. Thus, the elevated CAT activity in *P. afra* plants during the CAM-idling likely suggests the occurrence of photorespiration. It has been shown that some CAM species present a reduction in glycolate oxidase activity when they accumulate more malate, as a metabolic signal of photorespiration suppression [28,29]. As we observed a reduction in nocturnal organic acid (likely malic acid) accumulation with the progression of water stress, we supposed that photorespiration could be active during the CAM-idling status, contributing to keep oxidized the photosynthetic electron transport chain. The photorespiration could also provide CO_2_ for the C3 metabolism during the daylight, as recently proposed by Guralnick and colleagues [22] for the C4 plant *Portulaca grandiflora*.

Converse to the enzymatic scavengers, the water-soluble and lipid-soluble antioxidant capacity decreased with the severity of stress. Generally, these antioxidants are increased in plants exposed to the water stress. Our data indicate that the non-enzymatic activities would not be responsible for controlling ROS when plants are in CAM and CAM-idling. The antioxidant system would have a protective function against ROS in plants which mainly utilize the C3 pathway. In fact, the antioxidant capacity remained low also after the rewatering, when plants were full CAM and maintained an elevated enzymatic antioxidant activity. Thus, we hypothesized that the enzymatic antioxidant activity would represent the main defense against ROS when plants shift from C3 to CAM-idling under severe water stress and to CAM after rewatering.

The high activity of scavenger enzymes, especially under strong water stress, suggests an intense activity of other photochemical processes than CO_2_ fixation in sustaining the electron transport, as indicated by the high level of photochemical quenching (qL) [30]. The qL index started to increase soon after water stress imposition remaining elevated during the whole period of stress, despite Φ_PSII_ and Φ_NPQ_ reduction. With the progression of water stress, a gradual increase of quantum yield of non-regulated energy dissipation (Φ_NO_) was also observed. This condition usually suggests the occurrence of higher photo-damage risks and is related to the rise of ROS production [31]. Based on our results, we assumed that the rise of Φ_NO_ might represent a critical “regulatory” mechanism to guarantee the acclimation in *P. afra* under severe water stress, and more specifically under the CAM-idling status. On the other hand, the quantum yield of regulated energy dissipation (Φ_NPQ_) seems to have a role in counteracting the water stress at the first time, as previously demonstrated by Cela et al. [10].

This “regulatory” mechanism might operate together with PARP activity and potentiate the plant defenses during the CAM-idling status when the risks of oxidative damages are elevated. The safety role of PARP enzymes in plants has been widely proved in several C3 species in response to different stress, such as high temperature, ionizing radiation, and drought [13,32,33]. However, it is the first time that the function of poly (ADP) ribosylation is considered in a CAM-inducible species, and in particular under CAM-CAM-idling transition. In response to prolonged water stress, the PARP activity in *P. afra* plants was downregulated and this reduction would allow leaves to become more tolerant to severe water stress. The amplified tolerance might occur by either keeping the cell energy at a high level through a reduction in NAD^+^ breakdown and ATP overconsumption by PARP [13,14,34] or by ABA-dependent stress signaling [35]. The high NAD^+^ levels as a consequence of the reduced PARP activity, would lead to the synthesis of the cyclic ADP-ribose (cADPR) responsible for the abscisic acid (ABA) production and hence ABA-responsive gene expression. Further research studies are needed to understand the primary or ancillary role of the PARP enzyme in the tolerance to abiotic stress in CAM plants as well as in the seasonal shift from C3 to CAM photosynthesis. Data reported in this study seem to indicate a close relationship between the photosynthetic metabolic pathway and PARP activity. The poly (ADP-ribose) polymerase activity was higher after rewatering compared unstressed plants. It is likely due to the fact that under full CAM the potential for the oxidative stress was greater than under C3 metabolism, as consequence of the elevated electron transport activity (ETR) and internal O_2_ concentrations [2,3,4], which exposes plants to the oxidative stress. This would explain also the elevated activity of the enzymatic antioxidants under full CAM.

## 4. Materials and Methods

### 4.1. Plants Growth Condition and Experimental Design

**Figure 6:**
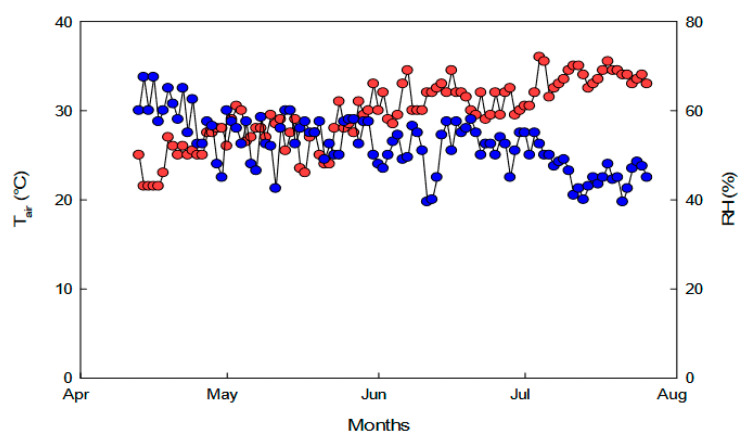
Daily average air temperature (red circles) and relative humidity (blue circles) inside the greenhouse during the experimental period.

Plants of *Portulacaria afra* (L.) Jacq. of three-years old were grown in a greenhouse under natural sunlight in pots of 5 L filled with a mixture of soil: peat (1:1 v:v) and irrigated to field water capacity. Ten plants were selected for water stress condition (water-stressed plants) and maintained without water up to 100 days (Days Without Water—DWW). Then, the stressed plants were watered for 14 days (RW—rewatering) to field water capacity to assess the plant capability to recover from stress. Trends of air temperature and relative humidity experienced by plants inside the greenhouse are shown in Figure 6.

The physiological behavior of *P. afra* plants during stress and rewatering was monitored at 7, 16, 27, 34, 56, 70, 85, and 100 DWW and after 14 RW by the measuring of titratable acidity and chlorophyll fluorescence emission. In addition, in some critical moments of the water stress period (16, 27, 56, and 100 DWW) and during the recovery (14 RW), the plant capability to withstand the stress was evaluated following the enzymatic and non-enzymatic antioxidants and poly(ADP-ribose)polymerase activity (PARP).

All the biochemical and physiological analyses were conducted on mature current-year leaves collected at midday, when *P. afra* plants were likely in the phase III of CAM. Five independent leaves per plant were chosen for analyses and the results were averaged. Statistical analysis was carried out on the averages.

### 4.2. Leaf Water Status and Photosynthetic Pigment Content Measurement

The leaf water status was assessed measuring the relative water content RWC = (FW − DW)/(TW − DW), where FW and TW are the fresh and turgid weight, respectively. DW represents the dry weight after oven-drying the leaves at 80 °C to constant weight. TW was determined under a vapor-saturated atmosphere dipping the petiole in water and keeping leaves darkened at 4 °C for 24 h.

Photosynthetic pigments (total chlorophylls and carotenoids) were extracted from samples collected at midday, in ice-cold 100% acetone with a mortar and pestle and centrifuged at 5000 rpm for 5’ (Labofuge GL, Heraeus Sepatech, Hanau, Germany). The absorbance of supernatants was quantified spectrophotometrically (UV-VIS Cary 100, Agilent Technologies, Santa Clara, CA, USA) at wavelengths of 470, 645, and 662 nm. The pigment content was calculated according to Lichtenthaler [36].

### 4.3. Titratable Acidity Determination

For the determination of titratable acidity, eight leaves per plant were collected at the early morning and the evening. Leaves from the same plant were mixed together and grounded in de-ionized water with a mortar and pestle and titrated with 0.01 N NaOH to a pH 7.0 endpoint. Data were expressed as μeq NaOH g^−1^ FW.

### 4.4. Chlorophyll a Fluorescence Emission Measurements

Chlorophyll *a* fluorescence measurements were carried out at midday by a pulse amplitude modulated fluorometer (Junior-PAM, Walz, Germany). Rapid light response curves (RLC) were performed on attached leaves at different light intensities (125–1500 μmol m^−2^ s^−1^) to assess the efficiency of the photosynthetic apparatus in the utilization of the absorbed light in photochemistry. Leaves were darkened for 30’ to determine the maximal PSII photochemical efficiency (F_v_/F_m_). The basal fluorescence signal (F_o_) was obtained by a weak beam of blue light at a frequency of 0.6 kHz, while the maximal fluorescence in the dark-adapted state (F_m_) was measured by applying a 1-s light saturating pulse. During exposure to light, the steady-state fluorescence (F_s_) was obtained by setting the measuring light to the frequency of 20 kHz; maximal fluorescence in the light-adapted state (F’_m_) was measured by applying a 1-s light saturating pulse.

The partitioning of absorbed light energy was estimated according to the model proposed by Kramer et al. [37]. The allocation of photons absorbed by the PSII antennae to photosynthetic electron transport was estimated as Φ_PSII_ = (F’_m_ − F_s_)/F’_m_. The quantum yield of regulated energy dissipation and of non-regulated energy dissipation within PSII were calculated as Φ_NPQ_ = 1 − Φ_PSII_ − 1/(NPQ + 1 + qL × (F_m_/F_0_ − 1)) and Φ_NO_ = 1/(NPQ + 1 + qL × (F_m_/F_0_ − 1)), respectively. The coefficient of photochemical quenching, qL, was defined and estimated following Kramer et al. [37]. The electron transport rate (ETR) was calculated according to Krall and Edwards [38] as Φ_PSII_ × PPFD × 0.5 × 0.84, considering the absorbed photon energy equally distributed between PSI and PSII and 0.84 the assumed light absorbance of the leaf.

### 4.5. Hydrogen Peroxide Determination

Among reactive oxygen species (ROS), the hydrogen peroxide (H_2_O_2_) is a constitutive product within cells and the variation in its concentration may suggest the occurrence of possible stress conditions. The H_2_O_2_ levels were measured following the colorimetric method described by Zhou et al. [39]. Briefly, leaves (0.3 g), frozen in liquid nitrogen, were grounded in a mortar with a pestle, in the presence of 3 mL of 5% TCA and 0.10 g activated charcoal. The homogenate was centrifuged at 10,000 rpm for 20 min at 4 °C, and the supernatant was added to 17 M ammonia solution and filtered. Then, a reaction mixture containing 1 mL of filtrate, 8 μg of catalase and 1 mL of colorimetric reagent was incubated for 10 min at 30 °C. The absorbance of the samples was determined spectrophotometrically at 505 nm. The colorimetric reagent contained 10 mg of 4-aminoantipyrine, 10 mg of phenol, 5 mg of peroxidase (150 U mg^−1^), dissolved in 50 mL of 100 mM acetic acid buffer (pH 5.6). The content of H_2_O_2_ was calculated using a standard curve and expressed as μmol H_2_O_2_ g^−1^ fresh weight (μmol H_2_O_2_ g^−1^ FW).

### 4.6. Non-Enzymatic and Enzymatic Antioxidant Measurements

The water-soluble antioxidants were extracted in a mixture consisting of methanol, water, and formic acid (80:20:0.1) for 3 h in the dark. The supernatant (S1), obtained after centrifugation at 3500 rpm for 1 min, at 4 °C, which represents the hydrophilic extract, was stored at 4 °C. The pellet was subjected to two subsequent extractions. The hydrophilic extracts S1, S2, and S3, were combined and further centrifuged at 10,000 rpm for 5 min at 4 °C. The lipid-soluble antioxidants were extracted in acetone by performing the same experimental procedure above described. The soluble and fat-soluble extracts were directly used in the antioxidant assay. Free radical scavenging activity of whole examined samples was determined by 2,2’-azino-bis(3-ethylbenzthiazoline-6-sulphonic acid) (ABTS^•+^) radical cation decolorization assay as described by Re et al. [40] with minor modification. ABTS^•+^ cation radical was obtained by the reaction between 7 mM ABTS in water and 2.45 mM potassium persulfate (1:1). The reaction mixture was stored for 16 h in the dark at room temperature before use and utilized within two days. The ABTS^•+^ solution was diluted with methanol, to obtain an absorbance of 0.700 ± 0.050 at 734 nm. Then 5 μL of plant extract was added to 3.995 mL of diluted ABTS^•+^ solution, and the absorbance was measured at 734 nm. An appropriate solvent blank was run in each assay. All the measurements were carried out at least three times. Trolox solution (final concentration 0–15 µM) was used as a reference standard. The results were expressed as µmol Trolox g^−1^ fresh weight of tissue.

Superoxide dismutase (SOD), Catalase (CAT), and Peroxidase (POD) activities were determined as reported in Arena et al. [15]. Briefly, SOD (EC 1.15.1.1) activity was determined by absorbance increase at 320 nm for 1 min. One unit of SOD activity is expressed as the amount of enzyme required to cause 50% inhibition of epinephrine oxidation under the experimental conditions. CAT (EC 1.11.1.6) activity assay was monitored as a decrease of H_2_O_2_ at 240 nm and quantified by its molar extinction coefficient (MEC = 36 M^−1^ cm^−1^). POD (EC 1.11.1.7) assay was determined by the increase in absorbance at 420 nm caused to guaiacol oxidation (MEC = 26.6 mM^−1^ cm^−1^).

### 4.7. Poly (ADP-Ribose) Polymerase (PARP) Assay Activity

The ADP-ribosylating activity was determined as reported in Arena et al. [15]. In brief, the assay was carried out on isolated nuclei incubated in presence of 0.4 mM [^32^P] NAD^+^ (10,000 cpm/nmole) in a 500 mM Tris-HCl buffer, pH 8.0; 50 mM MgCl_2_ and 10 mM DTT (reaction mixture). The reaction was stopped by adding ice-cold 20% trichloroacetic acid (*w*/*v*). Then, the mixture was filtered on Millipore filters (HAWPP0001, 0.45 μm) and subjected to various washes using 7% trichloroacetic acid. The radioactivity of insoluble acid material associated with the filter was measured in a liquid phase scintillator (Bechman LS 1701). The enzyme activity was expressed in enzymatic milliunit; 1 mU catalyzes the synthesis of 1 nmol ADP-ribose/min, at the optimal pH and temperature.

### 4.8. Statistical Analysis

Statistical analysis and graphical elaboration of the data were performed by of Sigma-Plot 12.0 software package (Jandel Scientific, San Rafael, CA, USA). Data were analyzed by one-way ANOVA repeated measurements followed by the Tukey’s test. The results are reported as mean (*n* = 10) ± SE. Differences were considered statistically significant at *p* ≤ 0.05. Shapiro–Wilk and Kolmogorov–Smirnov tests were performed to check for normality. The correlations between selected parameters were investigated using Pearson’s correlation test.

## 5. Conclusions

Our results showed that *P. afra* plants counteract the water stress by defense mechanisms operating both at the physiological and biochemical level. The scavenger enzymes and likely the photorespiration, dissipating the excess of absorbed light when the photosynthetic metabolism is down-regulated by severe water stress, preserve the photosynthetic apparatus integrity and guarantee a prompt recovery with the replacement of the optimal plant water status. With rewatering, the maximum photochemical efficiency is fully recovered, and H_2_O_2_ and Φ_NO_ are dropped to pre-stress values, confirming the relationship between hydrogen peroxide production and the non-regulated energy dissipation mechanisms. The regulation of PARP and antioxidant enzyme activity in synergy with the water-soluble and lipid-soluble antioxidant capacity and PSII photoprotective strategies, guarantees the tolerance of *P. afra* to severe and prolonged water stress. These strategies ensure a rapid physiological recovery with rewatering. Our results suggest that the PARP activity is modulated depending on the operating photosynthetic pathway.

## Figures and Tables

**Table 1:** Leaf relative water content (RWC) before (0 days) and during (60–94 days) water stress, and after the rewatering period of 14 day. Data are means (*n* = 10) ± SE.

Days Without Water (DWW)	Relative Water Content (%)
0	85.5 ± 2.0 ^a^
60	66.3 ± 4.3 ^b^
79	47.3 ± 3.16 ^c^
94	47.9 ± 4.1 ^c^
Days of Rewatering (RW)	RWC
14	88.9 ± 1.4 ^a^

Different letters indicate statistically significant differences (*p* ≤ 0.05) among days. Data were analyzed by one-way ANOVA repeated measurements followed by the Tukey’s test.

## Supplementary Materials

The following are available online at https://www.mdpi.com/2223-7747/9/9/1192/s1, Table S1: Coefficients of correlation (Pearson’s test) among the leaf investigated parameters.
