# Supplementary material for: Photo-Protective Mechanisms and the Role of Poly (ADP-Ribose) Polymerase Activity in a Facultative CAM Plant Exposed to Long-Term Water Deprivation
Source: Plants (Basel). 2020 Sep 12;9(9):1192. doi: 10.3390/plants9091192 (PMC7570031; doi:10.3390/plants9091192)
Supplement: Supplementary file 1 [file plants-09-01192-s001.pdf]

Table S1. Coefficients of correlation (Pearson's test) among the leaf investigated parameters: daily fluctuation of titratable acidity, TA; effective quantum yield of PSII photochemistry,Φ_{PSII}; regulated energy dissipation, Φ_{NPQ}; non-regulated energy dissipation, Φ_{NO}; electron transport rate, ETR; photochemical quenching, qL; maximum photochemical efficiency of PSII, F_v/F_m; hydrogen peroxide, H_2O_2; superoxide dismutase, SOD; peroxidase, POD; catalase, CAT; water-soluble antioxidants, WS-Antiox; lipo-soluble antioxidants, LS-Antiox; poly(ADP-Ribose) polymerase, PARP.

	**TA**	**Φ_{PSII}**	**Φ_{NPQ}**	**Φ_{NO}**	**ETR**	**qL**	**F_v/F_m**	**H_2O_2**	**SOD**	**POD**	**CAT**	**WS-Antiox**	**LS-Antiox**	**PARP**
**TA**	1	0.659	-0.361	-0.606	0.657	0.170	0.553	-0.445	0.355	0.368	0.404	-0.282	-0.243	0.257
**Φ_{PSII}**		1	-0.369	**-0.968**	**1**	-0.544	**0.974**	**-0.815**	-0.0326	-0.141	-0.095	0.283	0.401	-0.305
**Φ_{NPQ}**			1	0.125	-0.369	-0.196	-0.172	0.511	-0.256	-0.234	-0.237	0.452	0.332	-0.191
**Φ_{NO}**				1	**-0.968**	0.634	**-0.994**	0.732	0.104	0.214	0.165	-0.436	-0.534	0.132
**ETR**					1	-0.546	**-0.974**	**-0.816**	-0.0339	-0.143	-0.0964	0.284	0.403	-0.0764
**qL**						1	-0.689	0.468	0.462	0.517	0.488	-0.475	-0.580	0.326
**F_v/F_m**							1	-0.773	-0.149	-0.262	-0.217	0.455	0.570	-0.130
**H_2O_2**								1	0.462	0.517	0.488	-0.475	-0.580	-0.383
**SOD**									1	**0.980**	**0.987**	**-0.988**	**-0.967**	-0.507
**POD**										1	**0.997**	**-0.983**	**-0.970**	-0.364
**CAT**											1	**-0.975**	**-0.968**	-0.378
**WS-Antiox**												1	**0.973**	0.352
**LS-Antiox**													1	0.251
**PARP**														1

Data are means (n=10) ± SE. The significant Pearson's correlation coefficients are reported in bold ($p \leq 0.05$).
